# A Comparison of 2D and 3D Kidney Absorbed Dose Measures in Patients Receiving ^177^Lu-DOTATATE

**DOI:** 10.22038/aojnmb.2018.26105.1182

**Published:** 2018

**Authors:** Kathy P Willowson, HyunJu Ryu, Price Jackson, Anita Singh, Enid Eslick, Dale L Bailey

**Affiliations:** 1 Institute of Medical Physics, School of Physics, University of Sydney, Camperdown, NSW Australia; 2 Faculty of Health Sciences, University of Sydney, Cumberland, NSW Australia; 3 Department of Cancer Imaging, Peter MacCallum Cancer Centre, Melbourne, VIC Australia; 4 Department of Nuclear Medicine, Royal North Shore Hospital, St Leonards, NSW Australia

**Keywords:** Dosimetry, Kidney, Lutate, NET

## Abstract

**Objective(s):**

To investigate and compare quantitative accuracy of kidney absorbed dose measures made from both 2D and 3D imaging in patients receiving ^177^Lu-DOTATATE (Lutate) for treatment of neuroendocrine tumours (NETs).

**Methods:**

Patients receiving Lutate therapy underwent both whole body planar imaging and SPECT/CT imaging over the kidneys at time points 0.5, 4, 24, and 96-120 hours after injection. Planar data were corrected for attenuation using transmission data, and were converted to units of absolute activity via two methods, using either a calibration standard in the field of view or relative to pre-voiding image total counts. Hand drawn regions of interest were used to generate time activity curves and kidney absorbed dose estimates in OLINDA-EXM. Fully quantitative SPECT data were generated using CT-derived corrections for both scatter and attenuation, before correction for dead time and application of a camera specific sensitivity factor to convert data to units of absolute activity. Volumes of interest were defined for kidney using the co-registered x-ray CT, before time activity curves and absorbed dose measures were generated in OLINDA-EXM, both with and without corrections made to the model for patient specific kidney volumes. Quantitative SPECT data were also used to derive dose maps through dose kernel convolution (DKC), which was treated as the gold standard.

**Results:**

A total of 50 studies were analysed, corresponding to various cycles of treatment from 21 patients. Planar absorbed dose estimates were consistently higher than SPECT derived estimates by, on average, a factor of 3.

**Conclusion:**

Quantitative SPECT is considered the gold standard approach for organ specific dosimetry however often relies on in house software. As such planar methods for estimating absorbed dose are much more widely available, and in particular, are often the only source of reference in previously published data. For the case of Lutate dosimetry, planar measures may lead to a three-fold increase in measures of kidney absorbed dose.

## Introduction

^177^Lu -DOTATATE (Lutate) is fast developing as a promising treatment for neuroendocrine tumours (NETs). The treatment is a form of peptide receptor radionuclide therapy (PRRT) and is based on targeting of the somatostatin receptors expressed by NETs with the chelated peptide referred to as DOTATATE. The associated radiolabel, Lutetium-177 (^177^Lu), has a 6.7 day physical half-life and emits a beta particle with a maximum energy of 0.5 MeV for therapeutic purposes, as well as a primary gamma ray with energy 208 keV, ideal for imaging post-injection.

Due to clearance through the bladder and additional re-absorption by the proximal tubules, kidneys are expected to be the dose limiting organ when considering repeat treatment with Lutate, which is generally given as 4 individual cycles each spaced 8 weeks apart. Individual patient imaging plays a vital role in evaluation of disease targeting and, in particular, kidney absorbed dose estimates. Coupled with regular checks of patient glomerular filtration rate (GFR), kidney cumulative absorbed dose acts as a crucial indicator of a patient’s ability to manage further cycles of therapy. 

The currently accepted critical threshold of kidney absorbed dose is 23 Gy, which has an associated 5% probability of deterministic side effects within 5 years (1-3). However, these guidelines are based on dose thresholds extended from external beam radiotherapy (EBRT) (4), which is unlikely to be comparable to PRRT due to stark differences in radiation type, dose rate and heterogeneity of dose. More recent kidney toxicity thresholds of 28 Gy and 40 Gy (depending on the presence of additional risk factors ) have been proposed (5), and may prove to be adopted in the future after further studies. Attempts at defining absorbed dose measures and thresholds for toxicity based on Lutate treatment have traditionally been derived from 2D planar measures. Planar estimates of kidney absorbed dose have been reported by Kwekkeboom *et al* to be in the range of 0.88 mGy/MBq when accompanied by amino acid infusion (6), a figure which was later confirmed by Wehrmann *et al* with estimates of 0.9 mGy/MBq (7). Whilst planar imaging is a suitable means for evaluating treatment efficiency, it is not considered an accurate approach to image quantification, and so dosimetry. Even with appropriate estimates for correction of attenuation and scatter, planar data suffer from overlap of tissues and highly subjective organ definition, and will most likely lead to over-estimates of absorbed dose when compared to more accurate measures made from 3D imaging (1, 8). For example, Garkavij *et al* reported kidney dose measures made from conventional planar based methods to be 1.15 mGy/MBq compared to 0.81 mGy/MBq from 3D methods (8). The use of 3D data for dose estimates also has both user and method specific limitations. The use of models to estimate organ size as opposed to patient specific organ measures has been demonstrated to cause estimates of absorbed dose to differ by as much as twofold (9). Heikkonen *et al* also identified the effect that margin definition may have on 3D dose estimates, reporting a mean kidney dose of 0.44 mGy/MBq when delineating kidneys according to the anatomical boundary compared to 0.74 mGy/MBq when using a small sub-volume (10).

The gold standard for image quantification, namely 3D quantitative single photon emission computed tomography (SPECT)/CT, is not widely available and is generally based on specialised in-house software, utilising a wide variety of essential corrections (11). Recent measures of kidney absorbed dose from quantitative SPECT/CT have been made by Bailey *et al*, estimated at 12.0 Gy per treatment, where a treatment consists of 4 cycles of therapy, each of a nominal injected activity of ~8 GBq. This equates to approximately 0.4 mGy/MBq, based on average figures, which is lower than that reported by planar methods (12). Such estimates are also well below the accepted critical threshold for toxicity of 23 Gy. However, such methods for dosimetry are likely to be more time consuming, particularly with 3D imaging and organ segmentation required. As such, many centres performing Lutate will likely only have access to planar estimates of kidney absorbed dose to guide future therapy. In addition, given current trends towards future release of vendor specific quantitative SPECT software and dosimetry software using convolution techniques, it is likely that an understanding of translation of 2D estimates to equivalent 3D estimates will be needed to put existing recommended thresholds into context.

This study compares Lutate kidney absorbed dose measures made from both sub-optimal and optimal 2D and 3D data. The comparison represents a measure of error that centres might expect when using the simplified and more widely available approach of 2D planar assessment of kidney absorbed dose in patients receiving Lutate therapy for NETs.

## Methods

For Lutate treatments analysed in this paper, all preparations of non-carrier added ^177^Lu -DOTATATE followed the methods described in a recent paper from our institution by Aslani *et al* (13) and were accompanied by an amino acid infusion (Baxter Synthamin® Amino Acid (AA 10% - L-Lysine 5.8 g, L-Arginine 11.8 g in 500 mL)) given as 250 mL/hr for 30 mins prior and for 2 hours post cessation of lutate infusion (not stopped during lutate infusion), in order to reduce re-absorption of the ^177^Lu -DOTATATE in the proximal tubules and hence enhance clearance from the kidney to the bladder. 

Following injection of a standard amount of Lutate (~8 GBq), patients underwent imaging at 0.5 (20/50 studies), 4, 24 and 96-120 h, in the form of whole body planar acquisition and SPECT/CT acquisition, centred on the kidneys. All image data were acquired on a Siemens Intevo SPECT/CT system, with crystal thickness 16 mm, using a medium energy parallel hole collimator and a 20% energy window centred on 208 keV. Image acquisition protocols and reconstruction details are described in full in our previous publication (12). 

A total of five different dose estimates were made for each data set, two based on 2D approaches and three based on 3D approaches: a quantitative planar estimate; a semi-quantitative planar estimate; a model-based quantitative SPECT estimate; a patient-specific quantitative SPECT estimate; and a dose kernel convolution (DKC) estimate.

***Planar dosimetry***

**Figure 1:**
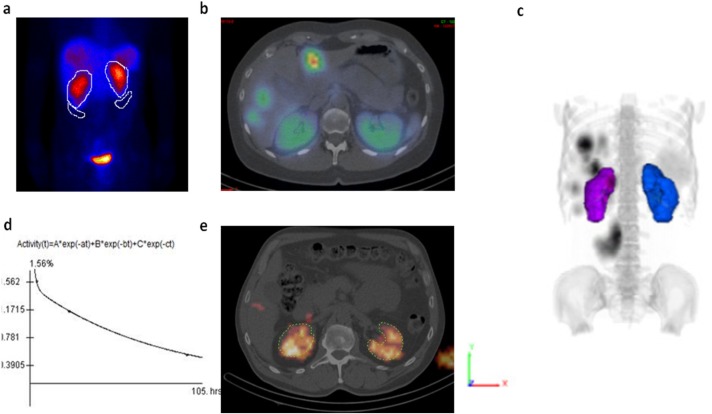
Analysis corresponding to each of the dosimetry methods used: planar ROIs on 2D data (a); CT-defined VOIs on 3D quantitative SPECT data (b,c); TACs generated in OLINDA-EXM to derive dose estimates from both 2D and 3D activity measures (d); and CT-defined VOIs on 3D dose maps (e

To produce quantitative planar images, methodology originally described in MIRD Pamphlet #16 (14) was implemented using in-house developed software using a high-level scientific programming language (IDL, Exelis Visual Information Solutions, Herndon, VA, USA) on a dedicated nuclear medicine workstation (HERMES, Nuclear Diagnostics, Stockholm, Sweden). Quantification included the use of blank and transmission images to perform broad beam attenuation correction, as well as the use of a calibrated standard in the field of view (FOV) to convert corrected counts in to units of absolute activity. An experienced nuclear medicine technologist (AS) defined regions of interest (ROIs) over both the right and left kidney which were propagated across all time points, with corrections for background based on background ROIs positioned inferior to the lower pole of each kidney (Figure 1a), and the total activity recorded. Time-activity curves (TACs) were generated for each study in OLINDA-EXM (15), utilising the adult male and female models where appropriate, and fitting with a mono-exponential led to absorbed dose estimates for kidney. 

A sub-optimal planar method was also used to generate semi-quantitative planar data, which involved masking of the standard on the 0.5 hour (pre-voiding) image for normalisation to the net injected activity. The associated derived sensitivity factor was then applied to all subsequent imaging time points. Identical kidney and background ROIs defined for the quantitative 2D data (above) were then applied, to derive TACs and generate sub-optimal planar imaging absorbed dose estimates in OLINDA-EXM for comparison with the quantitative 2D method.

**Table 1:** Summary of resulting kidney absorbed dose measures for each of the five dosimetry methods investigated

**Method**	**Mean (Gy/Cycle)**	**Mean (mGy/MBq)**	**Minimum (mGy/MBq)**	**Maximum (mGy/MBq)**
2D quantitative	8.27 ± 4.49	1.07 ± 0.58	0.35	2.94
2D semi-quantitative	8.18 ± 3.15	1.04 ± 0.39	0.44	1.73
3D model volumes	3.63 ± 1.19	0.47 ± 0.15	0.19	0.77
3D patient volumes	3.49 ± 1.25	0.45 ± 0.16	0.21	0.79
3D DKC	2.64 ± 1.09	0.34 ± 0.14	0.09	0.63

***SPECT dosimetry***

For 3D dose estimates, a previously developed in-house method for quantitative SPECT was used to generate SPECT data in units of absolute activity (12, 16). This included transmission based scatter correction, attenuation correction, dead time correcton, and application of a derived camera-specific sensitivity factor. Quantitative SPECT data were fused with the associated CT data and anatomical volumes of interest (VOIs) corresponding to kidney were defined and used to measure absolute activity at each time point (Figure 1b,c). All co-registration and VOI definition was performed using DOSIsoft^®^ software (Cachan, France). Once again, TACs were generated in OLINDA-EXM (Figure 1d) and absorbed dose estimates were derived for each study using the appropriate models (adult male and female phantoms). Measured kidney volumes defined on the CT data were compared to the OLINDA-EXM model for male and female kidneys, respectively, and evaluated for fluctuations between cycles. Dose estimates were then modified by updating the OLINDA-EXM model to include patient specific kidney mass, as opposed to the standard model kidney mass, to look at the associated impact on absorbed dose measures.

A third 3D absorbed dose estimate was also made from dose maps derived from the 3D quantitative SPECT data using purpose built DKC software fitting three-phase exponential time activity curves (1 uptake, 2 clearance) at the voxel level (17). The ^177^Lu derived dose maps were co-registered to the CT at the first imaging time point before VOIs were again defined and used to measure mean absorbed dose (Figure 1e). The 3D DKC estimates were treated as the gold standard in this comparative analysis.

## Results

A total of 50 studies were analysed corresponding to cycles from 21 different patients (9 females and 12 males). Of these studies only 20 were analysed with the 2D semi-quantitative method as the 0.5 h imaging time point (pre-voiding) was necessary for this analysis, and was discontinued for clinical reasons during the duration of data collection. Treating the 3D DKC estimates as the gold standard, the average kidney absorbed dose associated with a single cycle of Lutate treatment was 2.64±1.09 Gy, corresponding to 0.34±0.14 mGy/MBq. Table 1 compares the five methods of analysis. 

The average kidney volume measured across the cohort was 351±82 cm^3 ^for men and 269±45 cm^3^ for women, compared to the standard OLINDA-EXM model for males and females of 299 cm^3^ and 275 cm^3^, respectively. On average, the measured kidney volumes deviated from that assumed by OLINDA-EXM by 2.7%, with a maximum deviation of 43%. It was also noted that some patients had substantial fluctuations in measured kidney volumes between cycles of treatment, in some cases demonstrating up to a 40% difference. 

## Discussion

Estimates of kidney absorbed dose made from 2D imaging agree well with previous estimates in the literature reported to be in the range of 0.9–1.15 mGy/MBq (7, 8).Findings for 3D methods are also in agreement with formerly published estimates of 0.4 mGy/MBq (12), however lower than other estimates reported at 0.90 mGy/MBq (8). Dose values derived through the DKC method are further decreased, which may in part be due to the effects of blurring outside the anatomically defined volume from the action of convolution. This may be mitigated through use of a threshold or regional VOI only, as employed by Garkavij *et al* (8). However the automated DKC approach removes much of the subjective processing that is required when analysing quantitative SPECT data with OLINDA-EXM, and in addition is a faster more automated process, likely resulting in more consistent estimates. The DKC Lutate kidney dose estimates were treated as the gold standard in this work, and most likely indicate the direction where future processing systems for dosimetry are headed.

The use of a calibration standard in the FOV as opposed to normalisation of pre-voiding counts did not have a significant impact on the quantification of 2D planar whole body data for kidney dose estimates. The use of 3D quantitative SPECT/CT in conjunction with OLINDA-EXM produced dose estimates that were consistently lower than those derived through 2D methods. This is in agreement with existing observations (1, 8), and on average resulted in a 56% reduction in kidney absorbed dose measures. The incorporation of patient specific kidney volumes in to the OLINDA-EXM model, as opposed to the standard phantom volumes for males and females, reduced dose estimates even further. On average the resulting reduction in dose estimates was only 3%. However, in some patients with particularly large or small kidney volumes, the deviations were significant, with a maximum reduction of 43% found as a result of patient specific volume inclusion. Whilst not as large as previous findings on deviations (9) this suggests caution when using the standard phantom volumes for certain patients.

The use of DKC provided the lowest estimates of absorbed dose to kidney which were on average 27% lower than the standard 3D quantitative SPECT method, and 68% lower than the 2D quantitative method. This DKC technique employs a different pharmacokinetic modelling routine to that used in the 3D OLINDA-EXM estimates; incorporating an early uptake phase peaking at 1-2 h after injection. Given the sparse temporal data, the finding suggests that dose estimates are also sensitive to the curve-fitting algorithm which can optimise many different solutions based on a limited number of data points. 

Considering maximum deviation of absorbed dose between cycles for a given patient when using the DKC approach to dosimetry, the largest difference in our cohort was found to be 1.8 Gy (or 0.22 mGy/MBq), and the average maximum deviation between intra-patient cycles was found to be 0.96 Gy (or 0.11 mGy/MBq). The average intra-patient standard deviation was 0.059 mGy/MBq (equivalent to 0.47 Gy for a standard patient injection of 8 GBq). These findings give credit to the potential of using cycle 1 dosimetry to not only estimate absorbed dose associated with further cycles, yet also potentially manipulate injected activity for optimal patient response based on this assumption.

Given the consistent over-estimates of 2D planar measures of kidney absorbed dose, this suggests that there is potential for patients to be under-treated, if based on treating to maximum tolerable dose in kidneys, when this analysis technique is used to monitor cycles and advise on continuation of, or prescribed activity for, therapy. From our cohort that had received their full Lutate treatment, 6 of the 10 patients were found to have received greater than the acceptable threshold of 23 Gy when 2D quantitative planar imaging was used for dosimetry. In contrast, none of the patients had received greater than the 23 Gy threshold when using 3D analysis techniques. The fact that DKC measures reduce associated dose by, on average, 68% when compared to conventional 2D planar techniques, suggests that further studies may recommend increasing the activity administered to patients per cycle, or alternatively, increasing the number of cycles that may be offered for long term management of responding patients.

## Conclusion

Assessment of absorbed dose to kidney plays a vital role in maintaining safe delivery of Lutate therapy to patients suffering from NETs. Derivation of absorbed dose estimates made from 2D planar data consistently over-estimate absorbed dose by approximately three-fold. This over-estimate should be taken into account when patients approach or surpass the accepted cumulative dose threshold of 23 Gy at sites using 2D image based dosimetry for patient monitoring.

## Authors’ Contribution

KW contributed the study concept and design, as well as generation of quantitative data, dose maps, and performed analysis in DOSIsoft and OLINDA-EXM, as well as being the primary manuscript producer. HR and EE contributed to study design and generated quantitative data, performed analysis in both DOSIsoft and OLINDA-EXM and aided in manuscript preparation. AS provided expertise in analysis of 2D data and aided in manuscript preparation. PJ contributed the in-house software for generation of ^177^Lu dose maps through dose kernel convolution and aided in manuscript preparation. DB advised on study design and analysis techniques, and aided in manuscript preparation.

## References

[1] Sandstrom M, Garske U, Granberg D, Sundin A, Lundqvist H (2010). Individualized dosimetry in patients undergoing therapy with (177)Lu-DOTA-D-Phe(1)-Tyr(3)-octreotate. Eur J Nucl Med Mol Imaging.

[2] Valkema R, Pauwels SA, Kvols LK, Kwekkeboom DJ, Jamar F, de Jong M (2005). Long-term follow-up of renal function after peptide receptor radiation therapy with (90)Y-DOTA(0),Tyr(3)-octreotide and (177)Lu-DOTA(0), Tyr(3)-octreotate. J Nucl Med.

[3] Cremonesi M, Ferrari M, Bodei L, Tosi G, Paganelli G (2006). Dosimetry in peptide radionuclide receptor therapy: a review. J Nucl Med.

[4] Emami B, Lyman J, Brown A, Coia L, Goitein M, Munzenrider JE (1991). Tolerance of normal tissue to therapeutic irradiation. Int J Radiat Oncol Biol Phys.

[5] Bodel L, Cremonesi M, Ferrari M, Pacifici M, Grana CM, Bartolomei M (2008). Long-term evaluation of renal toxicity after peptide receptor radionuclide therapy with 90Y-DOTATOC and 177Lu-DOTATATE: the role of associated risk factors. Eur J Nucl Med Mol Imaging.

[6] Kwekkeboom DJ, Bakker WH, Kooij PP, Konijnenberg MW, Srinivasan A, Erion JL (2001). [177Lu-DOTAOTyr3]octreotate: comparison with [111In-DTPAo]octreotide in patients. Eur J Nucl Med.

[7] Wehrmann C, Senftleben S, Zachert C, Muller D, Baum RP (2007). Results of individual patient dosimetry in peptide receptor radionuclide therapy with 177Lu DOTA-TATE and 177Lu DOTA-NOC. Cancer Biother Radiopharm.

[8] Garkavij M, Nickel M, Sjogreen-Gleisner K, Ljunberg M, Ohlsson T, Wingardh K (2010). 177Lu-[DOTA0,Tyr3] octreotate therapy in patients with disseminated neuroendocrine tumours: Analysis of dosimetry with impact on future therapeutic strategy. Cancer.

[9] Pauwels S, Barone R, Walrand S, Borson-Chazot F, Valkema R, Kvols LK (2005). Practical dosimetry of peptide receptor radionuclide therapy with (90)Y-labeled somatostatin analogs. J Nucl Med.

[10] Heikkonen J, Mäenpää H, Hippeläinen E, Reijonen V, Tenhunen M (2016). Effect of calculation method on kidney dosimetry in 177Lu-octreotate treatment. Acta Oncol.

[11] Ljunberg M, Celler A, Konijnenberg MW, Eckerman K, Dewaraja YK, Sjogreen-Gleisner K (2016). MIRD pamphlet No 26: Joint EANM/MIRD guidelines for quantitative 177Lu SPECT applied for dosimetry of radiopharmaceutical therapy. J Nucl Med.

[12] Bailey D, Hennessy T, Willowson K, Schembri G, Roach P, Snowdon G (2015). Quantitative biodistribution & kinetics of a new formulation of [Lu-177]-Octreotate. J Nucl Med.

[13] Aslani A, Snowdon GM, Bailey DL, Schembri GP, Bailey EA, Pavlakis N (2015). Lutetium-177 DOTATATE Production with an Automated Radiopharmaceutical Synthesis System. Asia Ocean J Nucl Med Biol.

[14] Siegel JA, Thomas SR, Stubbs JB, Stabin MG, Hays MT, Koral KF (1999). MIRD pamphlet no 16: techniques for quantitative radiopharmaceutical biodistribution data acquisition and analysis for use in human radiation dose estimates. J Nucl Med.

[15] Stabin MG, Sparks RB, Crowe E (2005). OLINDA/EXM: the second-generation personal computer software for internal dose assessment in nuclear medicine. J Nucl Med.

[16] Willowson K, Bailey DL, Baldock C (2008). Quantitative SPECT reconstruction using CT-derived corrections. Phys Med Biol.

[17] Jackson PA, Beauregard JM, Hofman MS, Kron T, Hogg A, Hicks RJ (2013). An automated voxelized dosimetry tool for radionuclide therapy based on serial quantitative SPECT/CT imaging. Med Phys.

